# Effect of Pulmonary Rehabilitation on Postoperative Clinical Status in Patients with Lung Cancer and Chronic Obstructive Pulmonary Disease: A Systematic Review and Meta-Analysis

**DOI:** 10.1155/2022/4133237

**Published:** 2022-03-28

**Authors:** Lu Wang, Mingwei Yu, Yunfei Ma, Rong Tian, Xiaomin Wang

**Affiliations:** ^1^Beijing Hospital of Traditional Chinese Medicine, Capital Medical University, No. 23, Back Road of Art Gallery, Dongcheng District, Beijing 100010, China; ^2^Beijing Geriatric Hospital, No.118 Wenquan Road, Wenquan Town, Haidian District, Beijing 100095, China

## Abstract

Pulmonary rehabilitation (PR) has a curative effect in patients undergoing pneumonectomy for lung cancer. Nevertheless, the contribution of PR to the clinical status of patients with chronic obstructive pulmonary disease (COPD) undergoing lung resection has not been adequately elucidated. The aim of this systematic review of randomized and nonrandomized controlled trials was to appraise the impact of PR compared to conventional treatment based on postoperative clinical status in patients with lung cancer and COPD. Literature in English from PubMed, Cochrane Library, Science Citation Index, and Embase databases and in Chinese from the Chinese National Knowledge Infrastructure and the WANFANG Database was retrieved from inception to November 2021, employing the keywords “Pulmonary Neoplasms,” “Chronic Obstructive Pulmonary Diseases,” “Physical Therapy Modalities,” and “pulmonary rehabilitation.” Only studies that reported PR results were included. This review was registered in the International Prospective Register of Systematic Reviews (number: CRD42021224343). A total of nine controlled trials with 651 patients were included. Postoperative pulmonary complications (PPCs) were the primary outcome measure. PR decreased the risk of complications after surgery compared to regular treatment (odds ratio (OR) 0.21, 95% confidence interval (CI) 0.12–0.37, *P* < 0.01). PR reduced the risk of pneumonia after surgery compared to regular treatment (OR 0.36, 95% CI 0.15–0.86, *P*=0.02). There was a significant difference in the postoperative length of stay (mean difference −2.13 days, 95% CI −2.65 to −1.61 days, *P* < 0.05). PR was an effective intervention that decreased PPCs in patients suffering from lung cancer and COPD. However, due to the limitations of the available data, the results should be interpreted with caution.

## 1. Introduction

Many patients with non-small-cell lung cancer (NSCLC) have chronic obstructive pulmonary disease (COPD), which increases the risk of postoperative complications and mortality [1]. The incidence of lung cancer in COPD patients is higher than that in the general population [2]. Moreover, lung cancer is an important cause of morbidity and mortality in patients with COPD [3]. Moreover, COPD has been shown to be a leading cause of complications and postoperative recurrence of NSCLC [4]. The co-occurrence of lung cancer with COPD results in a poor prognosis. Therefore, patients with concurrent COPD and lung cancer should be paid special attention.

Surgery is the standard and most effective therapeutic approach for early-stage NSCLC [5]. The postoperative outcome of COPD patients with lung cancer is influenced by several complex factors. The decreased functional area in the lungs following resection may impose additional requirements on the organ. Most COPD patients have poor lung function and exercise capacity. Even if pneumonectomy is theoretically possible, some local functional differences may make surgery unfeasible for COPD patients [6], even at early-stage lung cancer.

The American Thoracic Society/European Respiratory Society Statement (2013) defines pulmonary rehabilitation (PR) as “a comprehensive intervention based on a thorough patient assessment followed by patient-tailored therapies, which include but are not limited to exercise training, education, and behavior change designed to improve the physical and psychological condition of people with chronic respiratory disease and to promote the long-term adherence of health-enhancing behaviors.” Pulmonary rehabilitation (PR) can reduce COPD-related dyspnea, enhance the quality of life, and shorten the hospitalization duration [7–9]. Patients with lung cancer often experience muscle weakness, deconditioning, fatigue, and anxiety, to which the negative influences of underlying COPD are added [10, 11]. Low exercise tolerance is associated with poor thoracic surgical outcomes and reduced survival among individuals with lung disease. PR has been shown to improve exercise tolerance and to be effective against dyspnea and fatigue [10, 12]. Nevertheless, the advantage of PR for the clinical status of postpneumonectomy COPD patients is unclear. A recent meta-analysis [13] showed that preoperative exercise training may decrease postoperative pulmonary complications (PPCs) in patients with lung cancer. However, the influence of exercise training in patients with COPD undergoing lung cancer resection has not been determined. Although PR is recommended for COPD, it is rarely used in clinical practice. At present, no high-quality studies have been conducted among patients with lung cancer who develop postoperative COPD. Thus, there is no clinical recommendation for patients with both COPD and lung cancer.

We conducted this meta-analysis with the aim of determining whether PR improves the postoperative clinical status in patients with both lung cancer and COPD.

## 2. Materials and Methods

### 2.1. Guideline Adherence

The present study adhered to the Preferred Reporting Items for Systematic Reviews and Meta-Analyses (PRISMA 2020) guidelines. The review was registered in the International Prospective Register of Systematic Reviews/PROSPERO (registration number: CRD42021224343).

### 2.2. Eligibility Criteria

The participants, intervention, comparison, and outcome (PICO) framework was used to determine the inclusion criteria. Therefore, studies which conformed to the following criteria were included in this review: (i) studies focused on COPD patients, diagnosed according to the Global Initiative for Obstructive Lung Disease diagnostic criteria, suffering from lung cancer and who underwent pulmonary lobectomy; (ii) studies with patients that received PR involving exercise therapy (e.g., breathing techniques, walking, and strength) with or without any form of education and/or self-management strategy; (iii) studies in which the control group received standard care (e.g., routine medicine and daily health guidance) without PR; and (iv) studies that reported at least one of the following parameters: PPCs summarized by type (pneumonia, atelectasis, pulmonary embolism, respiratory failure, dyspnea, hemorrhagic drainage, empyema, interstitial pneumonia, and bronchial fistula), cardiopulmonary exercise test results (VO_2_ peak), pulmonary function test (PFT) results (forced expiratory volume (FEV1), FEV1%, FEV1/forced vital capacity (FVC)), 6-min walking distance (6MWD), length of stay (LOS), adverse events, or health-related quality of life (i.e., St. George's Respiratory Questionnaire). PPCs were the primary outcome measure, as they are associated with higher mortality rates, higher hospital costs, and prolonged hospitalization length. Both randomized (RCT) and nonrandomized (non-RCT) studies were included to gain an overall understanding of the research field.

### 2.3. Search Methods

Studies published in English in PubMed, Cochrane Library, Science Citation Index, and Embase databases and in Chinese in the Chinese National Knowledge Infrastructure, and WANFANG database from inception to November 1, 2021, were retrieved without any limitation on country of origin or article type. The following combinations of search queries were used to retrieve the articles: (Lung cancer OR Lung neoplasms OR Pulmonary Neoplasms), (Pulmonary Disease, Chronic Obstructive OR Airflow Obstruction, Chronic OR COAD OR Chronic Obstructive Airway Disease OR Chronic Obstructive Pulmonary Diseases OR Chronic Obstructive Lung Disease), (Physical Therapy Modalities OR Physiotherapy OR Neurological Physiotherapy OR Neurophysiotherapy OR Physical Therapy OR Physical Therapy Techniques OR Physiotherapy), and (rehabilitation OR pulmonary rehabilitation OR respiratory rehabilitation OR lung rehabilitation OR lung therapy OR pulmonary treatment OR rehabilitation program). Search strategy details are provided in the Supplemental Materials-Search strategy.

### 2.4. Data Extraction

Two reviewers (LW and YFM) abstracted the characteristics of the retrieved studies. One reviewer extracted the following research features from the studies, while the other reviewer verified the accuracy of these features. The data extraction form comprised the following information: (i) methods, including study design, total duration of study, location, study setting, withdrawals, and date of the study; (ii) participants, including their number (recruited and completed), mean age, age range, sex, severity of condition, diagnostic criteria, and inclusion and exclusion criteria; (iii) intervention and comparison; (iv) primary and secondary outcomes specified and collected and time points reported; and (v) funding for studies and noticeable conflicts of interest of the authors who reported the studies. Differences were resolved by consensus or discussion involving a third reviewer (XMW). The data were transferred into Review Manager (RevMan version 5.3, The Cochrane Collaboration, London, UK). We compared the data in the systematic review with the studies and re-checked the data for accuracy.

### 2.5. Quality Assessment

Two reviewers (MWY and RT) assessed the risk of bias using the criteria outlined in the Cochrane Handbook for Systematic Reviews of Interventions. Disagreements, if any, were resolved by discussion or by involving another reviewer. The Cochrane Collaboration's risk-of-bias tool and the Newcastle–Ottawa Scale were used to assess the risk of bias in the evaluated randomized and nonrandomized studies.

### 2.6. Data Synthesis and Statistical Analysis

The meta-analysis was implemented by computing the effect size and 95% confidence intervals (CI) using the RevMan software. The research included RCT and non-RCT studies but only RCT studies were included on the meta-analysis. The effect estimate was presented as odds ratios (ORs) for the dichotomous outcomes (PPCs).

The mean difference (MD) was employed for continuous data (VO_2_ peak, PFT, 6MWD, and LOS) when results were measured identically between studies. The standardized mean difference (SMD) was used for combining trials measuring the same outcome but applying different methods. PPCs were evaluated using pooled ORs with corresponding 95% CIs, and a fixed-effect model was used to interpret potential clinical heterogeneity [14]. Statistical heterogeneity was estimated using the I^2^ statistic. High heterogeneity was defined as *I*^2^ >50%. Fixed- and random-effects models were used when *I*^2^ <50% or *I*^2^ >50%, respectively.

## 3. Results

### 3.1. Reports Identified

**Figure 1:**
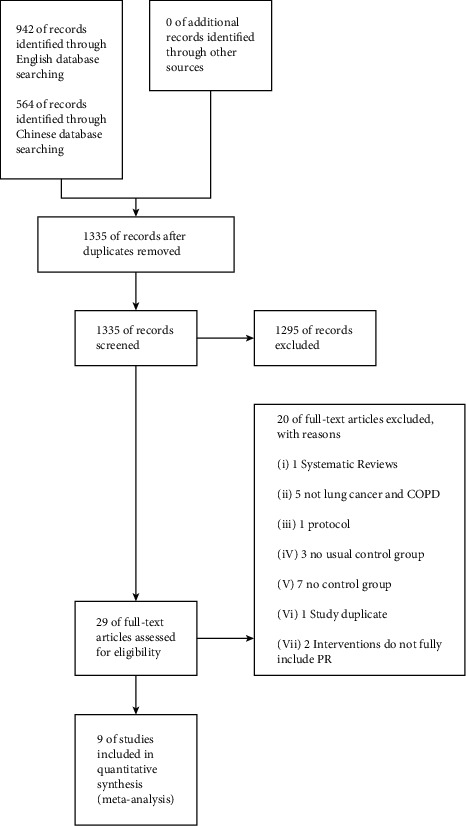
Flow diagram of study selection.

A total of 1335 records were initially identified. Twenty-nine articles were chosen for full-text review based on an initial review of the titles and abstracts of the identified articles. A total of nine articles [6, 15–22] were finally included in the study based on this full-text review. The flow diagram describing the literature search is presented in Figure 1.

### 3.2. Characteristics of the Included Studies

The characteristics of the included studies are shown in Table 1. All studies were published between 2005 and 2018. The countries of origin were Italy (*n* = 40) [16], China (*n* = 345) [15, 18–20], the USA (*n* = 19) [17], Japan (*n* = 144) [6, 20], and Serbia (*n* = 103) [21].

### 3.3. Participants

The included studies involved a total of 651 patients with lung cancer and COPD (age range: 50–81 years). The sample size of the controlled trials ranged from 19 to 110.

### 3.4. Intervention

The interventional designs are summarized in Table 2. The optimal form of exercise training for patients with lung disease has not been determined and may vary among individuals. However, the vast majority of studies and programs have used endurance training. Interval training and resistance/strength training have also shown benefits and could be utilized in combination with, or as a substitute for, endurance training. The protocol of all of the included studies included respiratory exercises and endurance training and the PR took place either daily [15, 17–20, 22] or for at least 5 days a week [6, 16, 21]. However, the length of intervention, duration and type of session, and intensity differed between studies. The length of intervention ranged from 1 to 4 weeks. Five research programs [16–20] mentioned training intensity.

### 3.5. Postoperative Pulmonary Complications

**Figure 2:**
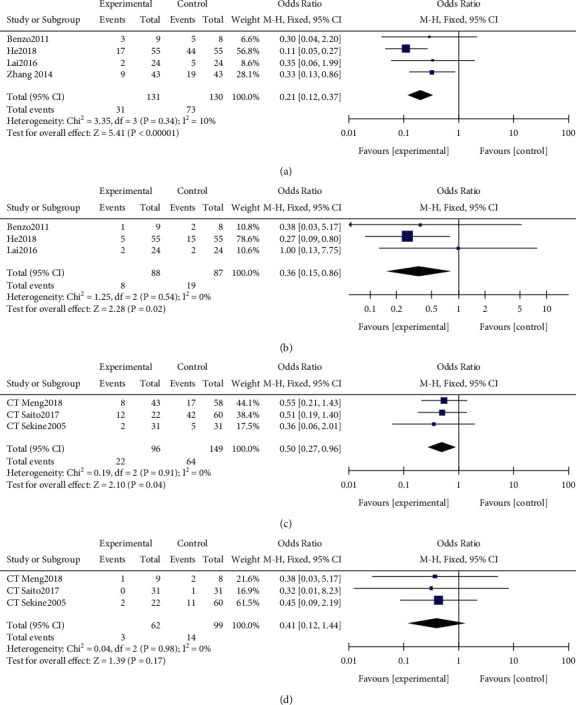
Forest plots comparing intervention and control groups in patients with lung cancer and chronic obstructive pulmonary disease (COPD) who were surgically treated. (a) Risk of postoperative pulmonary complications in randomized controlled trial (RCT). (b) Incidence of postoperative pneumonia in RCT. (c) Risk of postoperative pulmonary complications in computed tomography (CT) studies. (d) Incidence of postoperative pneumonia in CT studies.

Four RCT studies [17–20] reported PPCs. PR decreased the risk of postoperative complications (OR 0.21, 95% CI 0.12–0.37; Figure 2(a)), with acceptable heterogeneity (*I*^2^ = 10%, *P* < 0.01). PR reduced the risk of postoperative pneumonia (OR 0.36, 95% CI 0.15–0.86; Figure 2(b)), with acceptable heterogeneity (*I*^2^ = 0%, *P*=0.02). Three CT studies [6, 15, 22] reported PPCs. PR again decreased the risk of postoperative complications (OR 0.5, 95% CI 0.27–0.96; Figure 2(c)), with acceptable heterogeneity (*I*^2^ = 0%, *P*=0.04), but failed to reduce the risk of postoperative pneumonia (OR 0.41, 95% CI 0.12 1.44; Figure 2(d)). The difference was not statistically significant despite acceptable heterogeneity (*I*^2^ = 0%, *P*=0.17). Since it was unclear whether the researchers were blind to patient allocation to each group on this second set of studies, the potential for bias should be taken into account.

GRADE: the overall quality of this evidence is judged to be moderately downgraded, once for risk of bias and once for imprecision.

### 3.6. Cardiopulmonary Exercise Testing and Pulmonary Function Testing

**Figure 3:**
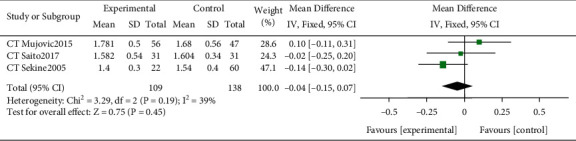
Forest plots of effect estimates of pulmonary rehabilitation (PR) versus controls on FEV1 (L).

Two RCT studies reported cardiopulmonary exercise test (CPET) and PFT results [16, 20]. Stefanelli et al. [16] compared CPET and PFT results after PR and 60 days after surgery. The other RCT study [20] compared postoperative CPET and PFT results. Since the test results were obtained at different time points, they could not be directly compared. Four CT studies [6, 15, 21, 22] reported results of PFT, but only in the study by Meng et al. [15], PFT results in the PR group before and after lung rehabilitation were compared. There were no significant differences in FEV1 (MD −0.04, 95% CI −0.15 to 0.07; Figure 3), and the heterogeneity of this result was acceptable (*I*^2^ = 39%, *P*=0.45).

GRADE: the overall quality of this evidence is judged to be very low, downgraded for risk of bias, imprecision, and the scarcity of data provided by the included studies.

### 3.7. Length of Stay

**Figure 4:**
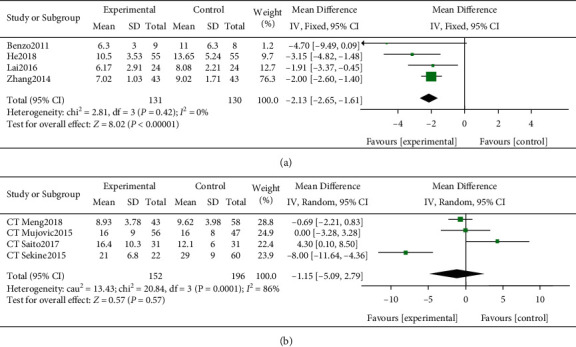
Forest plots of effect estimates of pulmonary rehabilitation (PR) versus controls on postoperative length of stay (LOS). (a) RCT studies that reported postoperative LOS. (b) CT studies that reported postoperative LOS.

Four RCT studies [17–20] reported LOS results. There were significant differences in postoperative LOS (MD −2.13 days, 95% CI −2.65 to −1.61 days; Figure 4(a)), and the heterogeneity of this result was acceptable (I^2^ = 0%, *P* < 0.05). Four CT studies [6, 15, 21, 22] also reported LOS results, but there were no significant differences in postoperative LOS (MD −1.15 days, 95% CI −5.09 to 2.79 days; Figure 4(b)), and the heterogeneity of this result was unacceptable (*I*^2^ = 86%, *P*=0.57).

GRADE: the overall quality of this evidence is judged to be low, downgraded once for risk of bias and once for imprecision.

### 3.8. Other Outcomes

Two RCT studies [18, 19] reported results for the 6-MWD test and only one RCT study [19] reported the result of health-related quality (no significant difference after PR). Only one CT study [21] reported the result of the 6-MWD test. One RCT study [17] reported on adverse events (no adverse effects were observed).

## 4. Discussion

The incidence of lung cancer is high, and COPD has been shown to worsen the prognosis for patients affected by this disease. The British Thoracic Society guidelines [23] recommend that COPD patients with lung cancer should undergo preoperative PR. It is unclear, however, whether PR can improve the postoperative condition of COPD patients with lung cancer. This meta-analysis included five RCTs and four CTs, totaling 651 participants in order to evaluate the evidence for an effect of PR in patients suffering from lung cancer and COPD.

The results of this meta-analysis suggested that, when compared to conventional treatment, PR may lower the incidence of PPCs in these patients. Our results are inconsistent with those of a previous meta-analysis [13]. Our meta-analysis included three studies [16, 18, 20] which were not included in the former meta-analysis, resulting in a larger sample size. The mechanisms by which PR improves PPCs remain unknown. However, muscle weakness and physical inactivity may participate in disease progression and may affect health-related quality of life, the frequency of pulmonary exacerbations, and the ability for mobilizing sputum [24]. Pehlivan et al. [25] have suggested that coughing, deep breathing, walking, and performing similar exercises might be conducive to a decreased incidence of pneumonia. Furthermore, differences in the definitions of complications between studies may have resulted in a bias when the PPCs were reported. To decrease this selection bias, we selected studies that precisely defined pneumonia as a normal complication, and we found that PR reduced the risk of postoperative pneumonia.

Moreover, the postoperative LOS was reduced after PR. LOS data from three studies [18–20] were not analyzed in the previous meta-analysis [13]. We failed to observe an improvement in lung function after PR, which is in accordance with the findings of the former meta-analysis [13].

Athletic ability and quality of life are two different areas of interest in rehabilitation research [26]. In patients with lung cancer, the improvement of exercise ability and quality of life may be more beneficial. Patients with chronic lung disease have been confirmed to benefit from PR. Theoretically, PR is presumed to improve pulmonary function and quality of life. A meta-analysis of 65 RCTs deduced that PR was more efficacious than normal community-based care in enhancing functional exercise capacity [9]. However, we found that there was less of difference in lung function between the PR and standard care groups. Three studies [18, 19, 21] in our meta-analysis reported 6MWD results, but due to differences in the research methods and the control groups, this variable could not be directly studied on the present meta-analysis. Some studies have demonstrated that PR improves the patient's quality of life [27, 28], but Lai et al. [19] reported no improvement after PR in their study. Few studies have drawn a clear conclusion regarding the subject, and future research should concentrate on exercise ability and quality of life in these patients.

Many researchers have corroborated the benefits of PR. It was believed that longer programs (e.g., 8 to 12 weeks) confer more enduring benefits and that they need to persist for at least 8 weeks to achieve a substantial effect [10, 29, 30]. However, impactful short-term preoperative PR projects are also relevant because patients with lung cancer often need to undergo surgery without delay. In nine studies, the PR duration was between 1 and 4 weeks. Some studies [31–33] demonstrated that postoperative PR improved exercise endurance and strength and reduced dyspnea in lung cancers. Saito et al. [6] designed a protocol for postoperative PR, whereas other studies did not include postoperative PR. Saito et al. [6] found that the FEV1 recovery rate one month after surgery was noticeably better in the PR group than in the control group. Thus, increasing the duration of a postoperative PR regimen may improve exercise capacity.

PR involves a combination of practices, including accurate diagnosis, therapy, emotional support, and education [34]. The protocols of the nine studies analyzed here included both respiratory exercises and endurance training. These protocols were adjusted according to the health status of the participants. Therefore, there is no consistent protocol for PR. Furthermore, the exercise and education components of PR are not always included in the protocols. Some studies [35–37] indicated that transcutaneous neuromuscular electrical stimulation or tai chi may be beneficial in PR. Tai chi and qi gong have been shown to be good substitutes for routine exercises during rehabilitation therapy [38]. Lu et al. [39] found that the combination of tai chi with traditional PR for lung cancer survivors was safe. It is therefore necessary to explore more effective rehabilitation treatments.

### 4.1. Limitations

There are some limitations to this study. First, significant heterogeneity was observed between studies. At present, there is no standard PR program. PR consists of exercise training, promotion of healthy behaviors, and psychological support. The protocols adopted by the nine included studies included exercise, but no one study involved promotion of healthy behaviors. Moreover, the length of intervention, duration of sessions, and exercise type and intensity differed. This could cause significant difficulties for the application of PR in a clinical setting, and highlights the need for a protocol with standard components, methods, intensity, and duration. Second, our meta-analysis included four non-RCT studies, which may have caused bias. Because of the particulars of PR, it is extremely difficult to perform a high-quality RCT to compare patients with lung cancer and COPD undergoing pneumonectomy. Since the implementation of a blind method is difficult to achieve, there was a high risk of bias in the five RCTs [16–20]. Finally, there is no objective index to evaluate the postoperative state, which affects the objective evaluation of PR. In addition, the criteria for evaluation of the postoperative state differ among studies.

### 4.2. Summary

In summary, patients with COPD and lung cancer are less likely to undergo surgery and tend to have a poorer postoperative status. PR was demonstrated to be an effective intervention that may reduce PPCs for patients with lung cancer and COPD. Clinicians may consider PR as a part of their treatment strategy according to their own clinical experience.

## Figures and Tables

**Table 1:** Characteristics of included studies.

Study (country)	Year	Study design	Total participants, *n* (male/female)	Intervention group (I), *n* (age, years); control group (C), *n* (age, years)	Characteristics	Comparison	Outcome measurements
Stefanelli et al. [16] (Italy)	2013	RCT	40 (23/17)	I: 20 (<75) C: 20 (75) 65 ± 7	NSCLC (stage I/II) and COPD; open thoracotomy	Usual care (preoperative preparation)	CPET PFT
Zhang and Zhao [17] (United States)	2011	RCT	19 (9/10)	I: 10 (70.2 ± 8.61) C: 9 (72.0 ± 6.69)	Lung cancer and COPD; open thoracotomy or VATS	Usual care (preoperative preparation)	PPCs LOS Adverse effects
Zhang et al. [18] (China)	2014	RCT	86 (56/30)	I: 43 (64.98 ± 2.7) C: 43 (64.53 ± 2.62)	NSCLC (stages I–III) and COPD; open thoracotomy or VATS	Usual care (preoperative preparation)	PPCs LOS 6MWD
Lai et al. [19] (China)	2016	RCT	48 (28/20)	I: 24 (63.13 ± 6.26) C: 24 (64.04 ± 8.94)	NSCLC (stages I–IV) and COPD; open thoracotomy or VATS	Usual care (preoperative preparation)	PPCs LOS 6MWD Health-related quality
He [20] (China)	2018	RCT	110 (72/38)	I: 55 (68.5 ± 5.6) C: 55 (69.3 ± 6.1)	NSCLC and COPD; open thoracotomy or VATS	Usual care (preoperative preparation)	PPCs LOS CPET PFT
Saito et al. [6] (Japan)	2017	CT	62 (54/8)	I: 31 (72.0 ± 8.8) C: 31 (71.3 ± 6.5)	NSCLC (stages I–II) and COPD; open thoracotomy or VATS	Usual care (preoperative preparation)	PPCs PFT LOS
Mujovic et al. [21] (Serbia)	2015	CT	103 (90/13)	I: 56 (72.0 ± 8.8) C: 47 (71.3 ± 6.5)	NSCLC and COPD; open thoracotomy or VATS	Usual care (preoperative preparation)	LOS PFT 6MWD
Sekine et al. [22] (Japan)	2005	CT	82 (76/6)	I: 22 (70.4 ± 4.6) C: 60 (69.0 ± 5.5)	NSCLC (stages I–IV) and COPD; Lobectomy	Usual care (preoperative preparation)	PPCs LOS PFT
Meng et al. [15] (China)	2018	CT	101 (63/38)	I: 43 (58.9 ± 8.9) C: 58 (61.1 ± 9.1)	NSCLC (stages I–III) and COPD; VATS	Usual care (preoperative preparation)	PPCs LOS PFT

RCT: randomized controlled trial; I: intervention group; C: control group; COPD: chronic obstructive pulmonary disease; SD: standard deviation; LOS: length of stay; NSCLC: non-small-cell lung cancer; PFT: pulmonary function testing; PPC: postoperative pulmonary complication; CPET: cardiopulmonary exercise test; 6MWD: 6-minute walking distance; VATS: video-assisted thoracoscopic surgery; CT: controlled trial.

**Table 2:** Summary of interventions.

Studies	Length of intervention	Duration of sessions	Type	Intensity	Frequency
Stefanelli et al. [16] (Italy)	3 weeks	3 hours	Lower limbs by treadmill and ergometric bicycle, respiratory exercises, and upper limbs with rowing ergometer	Started with 70% of the maximum score reached at the cardiopulmonary exercise test and increased by 10W when the patient was able to tolerate the set load for 30 min	5 per week
Benzo et al. [17] (United States)	1 week	15–20 minutes *∗* 3	Inspiratory muscle training, lower extremity endurance training, respiratory exercises, and strengthening exercises	If the patient perception was “too easy” or “requires no effort,” resistance was increased	Daily
Zhang and Zhao [18] (China)	2 weeks	35–50 minutes	Intense training (respiratory training and endurance training)	The exercise intensity was controlled within the target heart rate range	Daily
Lai et al. [19] (China)	1 week	15–30 minutes *∗* 4	Intense training (respiratory training and endurance training)	The amount of exercise was adjusted between Borg 5 and 7	Daily
He [20] (China)	2 weeks	15 minutes	Intense training (respiratory training and endurance training)	The amount of exercise was adjusted between Borg 5 and 7	Daily
Saito et al. [6] (Japan)	4 weeks		Peripheral muscle exercise training and respiratory exercise, postoperative PR	Not mentioned	5 per week
Mujovic et al. [21] (Serbia)	2–4 weeks	45 minutes	Intense training (respiratory training and endurance training)	Not mentioned	5 per week
Sekine et al. [22] (Japan)	2 weeks	30 minutes	Abdominal breathing and breathing exercises and walking more than 5,000 steps	Not mentioned	Daily
Meng et al. [15] (China)	7–10 days	20–30 minutes	Intense training (respiratory training and endurance training)	Not mentioned	Daily

## Data Availability

The datasets used and analyzed during the study can be obtained from the corresponding author upon reasonable request.

## Supplementary Materials

Supplementary Materials Appendix 1: MEDLINE (via PubMed) search strategy. Appendix 2: Cochrane Library search strategy. Appendix 3: Embase search strategy. Appendix 4: Science Citation Index search strategy. Appendix 5: Chinese National Knowledge Infrastructure search strategy. Appendix 6: WANFANG Database search strategy. Appendix 7: risk of bias.
