# Supplementary material for: Effect of Pulmonary Rehabilitation on Postoperative Clinical Status in Patients with Lung Cancer and Chronic Obstructive Pulmonary Disease: A Systematic Review and Meta-Analysis
Source: Evid Based Complement Alternat Med. 2022 Mar 28;2022:4133237. doi: 10.1155/2022/4133237 (PMC9122671; doi:10.1155/2022/4133237)
Supplement: Supplementary Materials — Appendix 1: MEDLINE (via PubMed) search strategy. Appendix 2: Cochrane Library search strategy. Appendix 3: Embase search strategy. Appendix 4: Science Citation Index search strategy. Appendix 5: Chinese National Knowledge Infrastructure search strategy. Appendix 6: WANFANG Database search strategy. Appendix 7: risk of bias. [file 4133237.f1.docx]

**Appendix 1. MEDLINE (via PubMed) search strategy**

#1 Search: (((Lung Neoplasms[MeSH Terms]) OR (Lung Cancer[Title/Abstract])) OR (Pulmonary Neoplasms[Title/Abstract])) OR (Lung Cancer[Title/Abstract])

#2 Search: (((((Pulmonary Disease, Chronic Obstructive[MeSH Terms]) OR (Airflow Obstruction, Chronic[Title/Abstract])) OR (COAD[Title/Abstract])) OR (Chronic Obstructive Airway Disease[Title/Abstract])) OR (Chronic Obstructive Pulmonary Diseases[Title/Abstract])) OR (Chronic Obstructive Lung Disease[Title/Abstract])

#3 Search: ((((((Physical Therapy Modalities[MeSH Terms]) OR (Group Physiotherapy[Title/Abstract])) OR (Neurological Physiotherapy[Title/Abstract])) OR (Neurophysiotherapy[Title/Abstract])) OR (Physical Therapy[Title/Abstract])) OR (Physical Therapy Techniques[Title/Abstract])) OR (Physiotherapy[Title/Abstract])

#4 Search: ((((((rehabilitation[MeSH Terms]) OR (pulmonary rehabilitation[Title/Abstract])) OR (respiratory rehabilitation[Title/Abstract])) OR (lung rehabilitation[Title/Abstract])) OR (lung therapy[Title/Abstract])) OR (pulmonary treatment[Title/Abstract])) OR (rehabilitation program[Title/Abstract])

#5 #3 OR #4

#6 #1 AND #2 AND #5

**Appendix 2. Cochrane Library search strategy**

#1 MeSH descriptor Lung Neoplasms, this term only

#2 MeSH descriptor Carcinoma, Non-Small-Cell Lung, this term only

#3 (lung cancer*):ti,ab,kw

#4 (non-small cell):ti,ab

#5 (small cell):ti,ab

#6(#1 OR #2 OR #3 OR #4 OR #5)

#7 (Chronic obstructive pulmonary disease)：ti,ab

#8 (COPD)：ti,ab

#9 (Chronic obstructive airway disease)：ti,ab

#10(Chronic obstructive respiratory disease)：ti,ab,

#11 MeSH descriptor Pulmonary Disease, Chronic Obstructive, this term only

#12 (#7 OR #8 OR #9 OR #10 OR #11)

#13 (rehabilitat*):ti,ab

#14 (lung therapy):ti,ab

#15 (pulmonary treatment):ti,ab

#16 MeSH descriptor Physical Therapy Modalities，this term only

#17 (#13 OR #14 OR#15 OR #16)

#18 (#6 AND #12 AND #17)

**Appendix 3. Embase search search strategy**

#1. 'lung cancer':ti,ab,kw

#2 'non-small cell':ti,ab,kw

#3 'small cell':ti,ab,kw

#4 'lung tumor'/exp

#5 #1 OR #2 OR #3 OR #4

#6 'chronic obstructive pulmonary disease':ti,ab,kw

#7 'copd':ti,ab,kw

#8 'chronic obstructive airway disease':ti,ab,kw

#9 'chronic obstructive respiratory disease':ti,ab,kw

#10 'chronic obstructive lung disease'/exp

#11 #6 OR #7 OR #8 OR #9 OR #10

#12 'rehabilitat*':ti,ab,kw

#13 'lung therapy':ti,ab,kw

#14 'pulmonary treatment':ti,ab,kw

#15 'chronic obstructive respiratory disease':ti,ab,kw

#16 'physiotherapy'/exp

#17 #12 OR #13 OR #14 OR #15 OR #16

#18 #5 AND #11 OR #17

**Appendix 4. Science Citation Index search strategy**


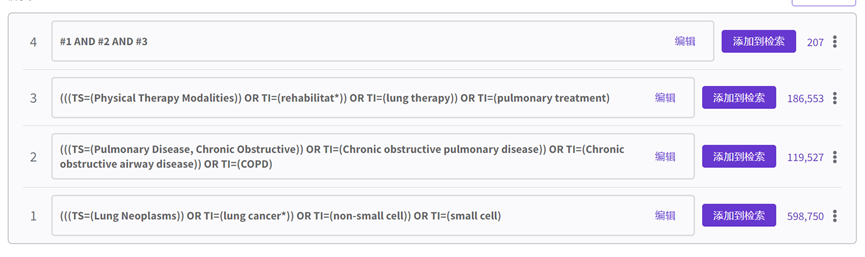


**Appendix 5. Chinese National Knowledge Infrastructure search strategy**

(SU=肺疾病，慢性阻塞性 OR SU=慢性阻塞性肺疾病)AND(SU=肺肿瘤 OR SU=癌，非小细胞 OR SU=癌，小细胞 OR KY=肺癌 OR TI=肺癌)484

AND（SU=肺切除术 OR TI=肺癌术后）AND（SU=物理治疗方法 OR SU=康复）

**Appendix 6. WANFANG Database search strategy**


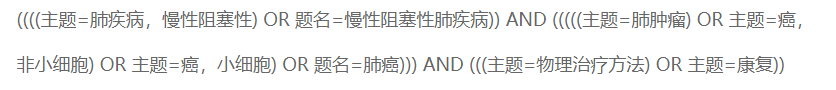


OR
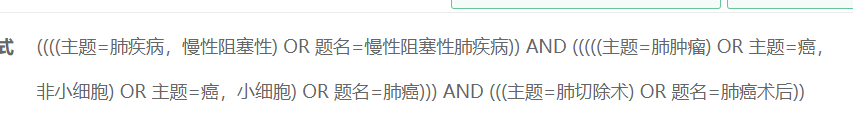


**Appendix 7. Risk of bias**


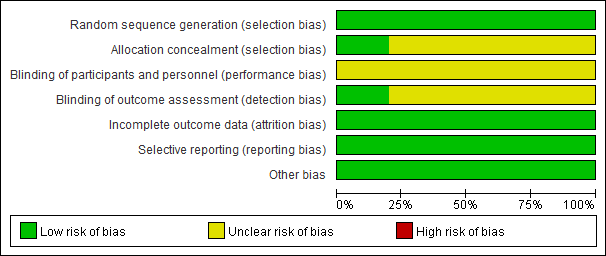


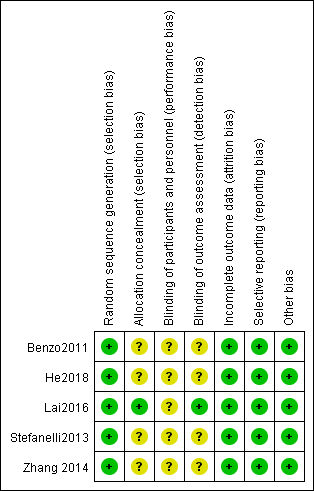


Newcastle–Ottawa Scale for CT

| Studies | Research object selection | Inter group comparability | Result factor measurement | Score |
| --- | --- | --- | --- | --- |
| Saito 2017 | 4 | 1 | 3 | 8 |
| Mujovic2015 | 4 | 1 | 2 | 7 |
| Sekine2005 | 4 | 1 | 2 | 7 |
| Meng 2018 | 4 | 1 | 2 | 7 |
